# High-Sensitivity Slot-Loaded Microstrip Patch Antenna for Sensing Microliter-Volume Liquid Chemicals with High Relative Permittivity and High Loss Tangent

**DOI:** 10.3390/s22249748

**Published:** 2022-12-12

**Authors:** Junho Yeo, Jong-Ig Lee

**Affiliations:** 1 School of Artificial Intelligence, Daegu University, Gyeongsan 38453, Republic of Korea; 2 Department of Electronics Engineering, Dongseo University, Busan 47011, Republic of Korea

**Keywords:** slot-loaded microstrip patch antenna, single-ring split ring resonator (SR-CSRR), polar liquid, high loss tangent, microliter-volume

## Abstract

This paper proposes a microwave sensor based on a high-sensitivity slot-loaded rectangular microstrip patch antenna (MPA) for measuring microliter-volume liquid chemicals with high relative permittivity and high loss tangent. A rectangular single-ring complementary split ring resonator (SR-CSRR) slot with a bottom-edge center split (BCS) was inserted along the upper radiating edge of the patch to enhance the relative permittivity sensitivity of the MPA. The first resonant frequency of the proposed SR-CSRR-BCS slot-loaded MPA showed the highest sensitivity compared to the resonant frequencies of the MPAs with other commonly used slots for varying the relative permittivity of the planar substrate type material under test from 1 to 10 when placed above the patch. After designing the scaled SR-CSRR-BCS slot-loaded MPA with the unloaded first resonant frequency at 2.5 GHz, a hollow acrylic cylindrical liquid container with an inner volume of approximately 18.6 μL was placed at the top-edge center of the SR-CSRR-BCS slot to achieve maximum sensitivity. A quarter-wavelength transformer was applied between the patch and the feed line of the MPA to improve the impedance mismatch that occurs when liquid chemicals with a high loss tangent are placed in the container. Water, methanol, and ethanol were carefully selected for test liquids to cover a broad range of relative permittivity and high loss tangents. The proposed SR-CSRR-BCS slot-loaded MPA was designed and fabricated on a 0.76 mm-thick RF-35 substrate, and a reference RS-loaded MPA was designed and fabricated for comparison. The shift in the first resonant frequency of the input reflection coefficient characteristic was used for the sensitivity comparison, and the container was filled with 15 μL of the liquids at 25 °C. The measured sensitivity (%) of the proposed SR-CSRR-BCS slot-loaded MPA for water was 0.45%, which was higher than other antenna-based microwave sensors in the literature.

## 1. Introduction

Over the past few decades, material characterization has been studied widely in various fields of science, engineering, and industry. In general, the electromagnetic properties of materials can be characterized by the electrical permittivity, electrical conductivity, and magnetic permeability [1]. Among the three electromagnetic parameters, the relative permittivity or dielectric constant of the material has been used to characterize the response of the material when the electric field is applied. The relative permittivity of a material is closely related to other physical and chemical properties, and can provide crucial information for various applications. Microwave-based permittivity measurement methods can be classified into resonant and non-resonant [2]. Variations in the characteristic impedance and wave velocity of electromagnetic waves are used to derive the permittivity of the material for non-resonant methods, such as a free space method or transmission line methods using a waveguide, a coaxial, a microstrip, and a coplanar waveguide. Changes in the resonant frequency and quality factor of the resonators are used in the resonant methods.

Because a liquid material has a fixed volume with no fixed shape, the permittivity of the liquid material is more susceptible to factors such as temperature, pressure, moisture, and contaminants than a solid material [3,4]. A liquid material can be classified as polar or non-polar [5]. A polar liquid consists of molecules with permanent dipole moments, whereas a non-polar liquid has molecules with zero dipole moments. A polar liquid has high relative permittivity in the range of 10 to 100 at radio and microwave frequencies because of the permanent dipoles in the molecules. It also has high dielectric loss at microwave frequencies with loss tangent ranges of 0.1 to 1. Examples of polar liquids include acetone, acetonitrile, dimethylformamide (DMF), dimethylsulfoxide (DMSO), ethanol, formic acid, glycerol, isopropanol, methanol, and water [6]. On the other hand, the relative permittivity and loss tangent of a non-polar liquid are relatively low, ranging from 2 to 3 and less than 0.001, respectively. Non-polar liquids include aniline, benzene, chloroform, cyclohexane, diethyl ether, ethyl acetate, hexane, pyridine, and toluene [6].

Recently, planar microwave resonators on various planar transmission lines have been investigated extensively to characterize a liquid material [7]. Commonly used structures for the planar resonators in the literature are a split-ring resonator (SRR), a complementary SRR (CSRR), and a complementary spiral resonator (CSR) [4]. An SRR is placed close to the microstrip line, whereas a CSRR and a CSR are placed on the ground plane of the microstrip line or the central line of a coplanar waveguide (CPW) line. Various types of SRR structures have been studied for the measurement of liquids. A microfluidic sensor consisting of a single SRR coupled to a microstrip transmission line was proposed for measuring liquid binary mixtures, such as ethanol–water and methanol–water [8]. Complex permittivity of ethanol–water and methanol–water mixtures at 1.9 GHz were extracted. A microwave microfluidic sensor based on a symmetric splitter/combiner configuration loaded with a pair of SRRs and microfluidic channels placed on top of the gap regions of the SRRs was introduced for the dielectric characterization of ethanol–water mixtures [9]. A glucose concentration sensor composed of a folded open-loop resonator or an SRR coupled to two microstrip lines was presented for microliter-volume water–glucose solutions [10]. Three versions of the sensor with different resonant frequencies between 2 to 7 GHz were introduced. Two models of liquid sample holders with 5 and 25 μL were fabricated from polytetrafluoroethylene (PTFE). A dual-sensing and dual-frequency microwave sensor consisting of two SRRs with different dimensions placed in the inner part of the microstrip power divider branches was proposed to detect liquids, such as ethanol, methanol, glucose solution, and water [11]. The relative permittivity of two different liquid samples can be detected at two separate frequencies simultaneously, and the relative permittivity of one liquid sample can be measured at two frequency bands.

A variety of CSRR-based microwave liquid sensors have been explored. A microwave sensor consisting of a folded microstrip transmission line with a middle square conductor and a CSRR on the ground plane was proposed for the dielectric characterization of binary mixtures of ethanol and water [12]. The liquid sample fills the glass capillary tube placed in a hole in the center of the CSRR and the square conductor. A square-shaped meander open CSRR (MOCSRR) in a CPW transmission line was used as a microwave sensor to analyze the dielectric properties of ethanol and water mixtures [13]. A liquid sample slot container using an FR4 substrate was used. An ultrahigh sensitivity microwave sensor consisting of a microstrip line loaded with a modified CSRR on the ground plane was introduced for liquid samples of ethanol–water solutions [14]. The microstrip line was modified to reduce the notch magnitude, whereas the meander slot was added in the single-ring CSRR to enhance the field confinement. A liquid-sensing low-cost microwave sensor with a triple-ring CSRR on the ground plane was proposed [15]. Two sets of sensors were designed at 1.2 and 2.4 GHz, and eight different liquid samples, hexane, acetic acid, acetone, methanol, acetonitrile, formic acid, hydrogen peroxide, and distilled water, were used.

Several types of CSR structures were developed for liquid-sensing microwave sensors. A microwave sensor consisting of a microstrip transmission line and a complementary circular spiral resonator etched on the ground plane was proposed to estimate the relative permittivity of ethanol–water mixtures [16]. A flexible microwave sensor composed of a two-turn CSR in the center line of a CPW line on a polyethylene terephthalate (PET) substrate was developed to be attached conformally to the palm of a dexterous robotic hand for use as a robotic electronic skin sensor [17].

Because a resonant antenna acts as a resonator, antenna-based microwave sensors have been investigated extensively for various sensing applications, such as permittivity/permeability, temperature, relative humidity, cracks, moisture, gas, impurities, and strain [18]. Among the various resonant antenna configurations, a microstrip patch antenna has been used widely for sensing applications because of its advantages, such as low profile, low cost, and easy fabrication. The first microstrip patch antenna used as a sensor was a coaxial-fed circular patch antenna for measuring the moisture content of sludge samples inside a plastic beaker placed on top of a circular patch [19]. Sludge samples were collected from a sewage treatment plant, and the variations in the resonant frequency of the dominant mode were used to determine the relative permittivity and moisture content using the particulate and Bottcher models. A coaxial-fed air-spaced rectangular microstrip patch antenna was proposed for the permittivity measurement of liquids, such as cyclohexane, pentan-1-ol, butan-1-ol, ethanol, and water [20]. The patch was considered buried in the liquid under the test, and the effective permittivity was calculated using the equation for a buried microstrip line. The effect of the moisture content on the resonant frequency shift of a rectangular microstrip patch antenna was studied when a liquid sample holder with Hevea rubber latex was placed above the patch [21]. A rectangular microstrip patch antenna with a double-ring CSRR slot on the patch was proposed as a microfluidic ethanol chemical sensor [22]. The microfluidic channel engraved on polydimethylsiloxane (PDMS) was placed above the CSRR. A rectangular microstrip patch antenna with a liquid chamber between the silicon substrates of the patch and the ground plane was proposed for measuring the salinities in seawater [23]. A slot-loaded microstrip patch antenna integrated with a microfluidic channel etched into the substrate between the patch and the ground plane was introduced for monitoring the moisture content of lubricating oil [24]. An edge-fed rectangular patch antenna with two T-shaped slots on the patch and a rectangular slab above the top radiating edge of the patch was proposed for wireless ice and frost detection applications [25].

Antenna structures other than the microstrip patch antenna were also studied for sensing liquids. A monopole antenna was used to measure the water content in soil and snow in the frequency range from 100 MHz to 2 GHz [26]. A U-shaped planar monopole antenna was proposed to measure aqueous glucose from 10 mg/mL to 40 mg/mL [27]. An eight-mode substrate-integrated-waveguide antenna (EMSIWA) with a microfluidic channel was introduced to measure the ethanol concentrations from 0% to 100% [28]. A crescent-shaped planar monopole antenna was used to detect salt and sugar in water [29]. A cylindrical dielectric resonator antenna (DRA) sensor was proposed to identify liquid chemicals, such as isopropyl, ethanol, methanol, and water [30]. A log periodic meandered dipole array antenna loaded with SRRs was introduced to detect different adulteration cases in liquids, such as dilutions of milk with water, adulteration of coconut oil with rice bran oil, adulteration of honey with sugar syrup, and various concentrations of salt and sugar in water [31]. A microstrip line-fed rectangular patch-shaped monopole antenna was used to detect ethanol concentration in wine and isopropyl alcohol in disinfectant [32]. A CPW-fed circular slot antenna with an electromagnetic bandgap (EBG) structure was proposed to detect liquids, such as butan-1-ol, propan-2-ol, ethanol, and methanol [33].

In this paper, a high-sensitivity rectangular microstrip patch antenna (MPA) loaded with a rectangular single-ring CSRR (SR-CSRR) slot with the split position in the bottom-edge center (BCS) of the ring is proposed to measure the microliter-volume liquid chemicals with high relative permittivity and high loss tangent. First, the mode characteristics and sensitivity of the first four resonant frequencies for the proposed SR-CSRR-BCS slot-loaded MPA were compared with the MPAs of other commonly used slots [34]. A hollow acrylic cylindrical liquid container with a volume of approximately 18.6 μL was placed at the top-edge center of the SR-CSRR-BCS slot for the scaled MPA, with the first resonant frequency at 2.5 GHz to achieve maximum sensitivity. A quarter-wavelength transformer was appended onto the feed line of the MPA to improve the impedance mismatch that occurs when liquid chemicals with high loss tangents are placed in the container. The scaled SR-CSRR BCS slot-loaded MPA with the quarter-wavelength transformer was designed and fabricated on a 0.76 mm-thick RF-35 substrate to have the first resonant frequency at 2.5 GHz under unloaded conditions. All the simulated results in this paper were obtained using CST Studio Suite (Dassault Systèmes Co., Vélizy-Villacoublay, France) [35].

## 2. Analysis of Reference Rectangular and Four Different Slot-Loaded MPAs

**Figure 1:**
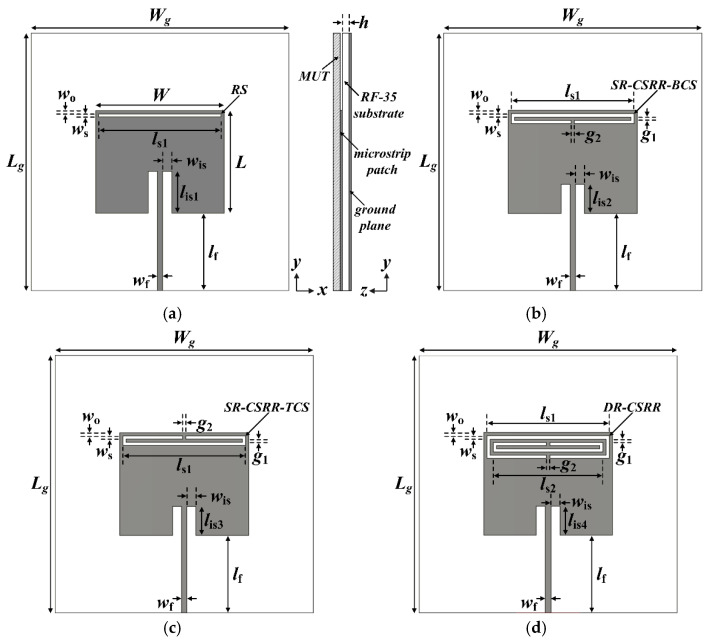
Geometries of the rectangular MPAs loaded with (**a**) a rectangular slot (RS), (**b**) a single-ring complementary split ring resonator with a bottom-edge center split (SR-CSRR BCS) slot, (**c**) an SR-CSRR with a top-edge center split (SR-CSRR TCS) slot, and (**d**) a double-ring CSRR (DR-CSRR) slot.

**Figure 2:**
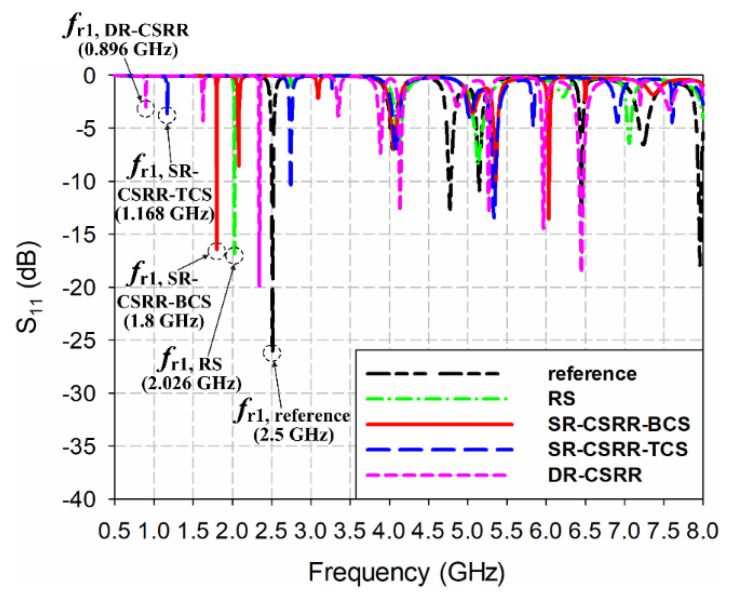
Comparison of the input reflection coefficient characteristics for the reference rectangular MPA with the four MPAs loaded with different slots.

**Figure 3:**
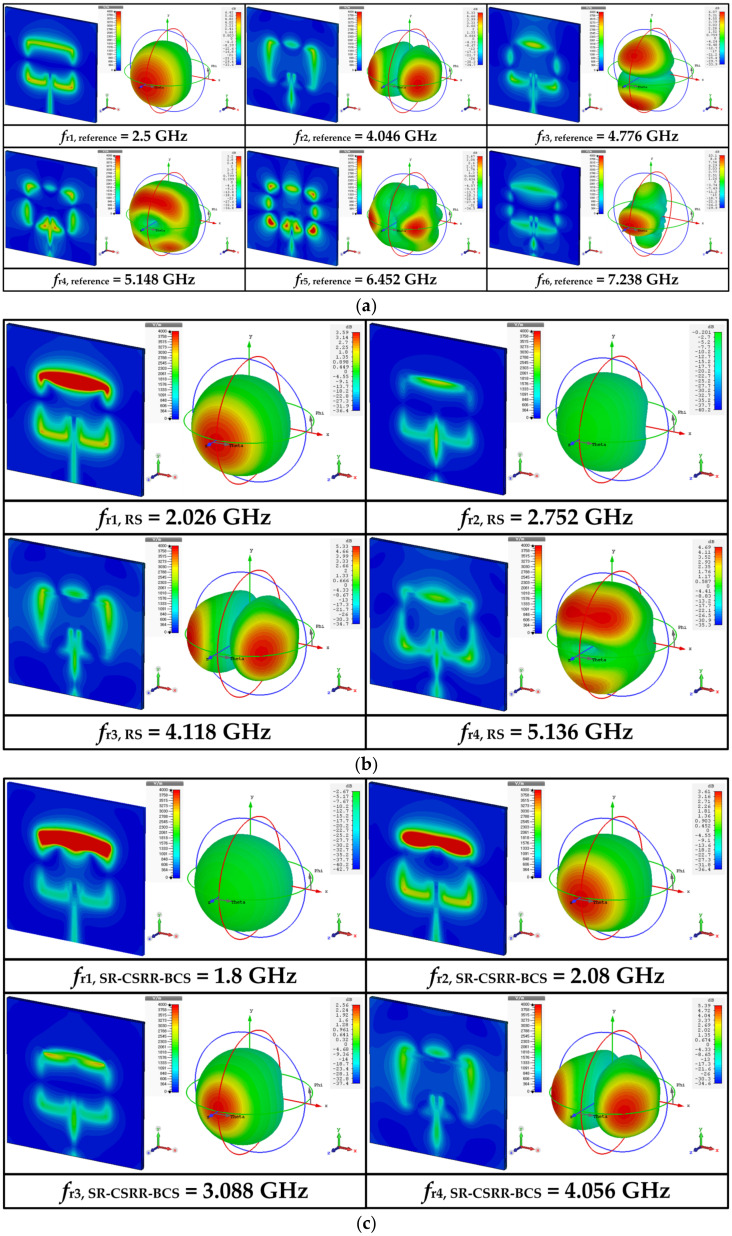

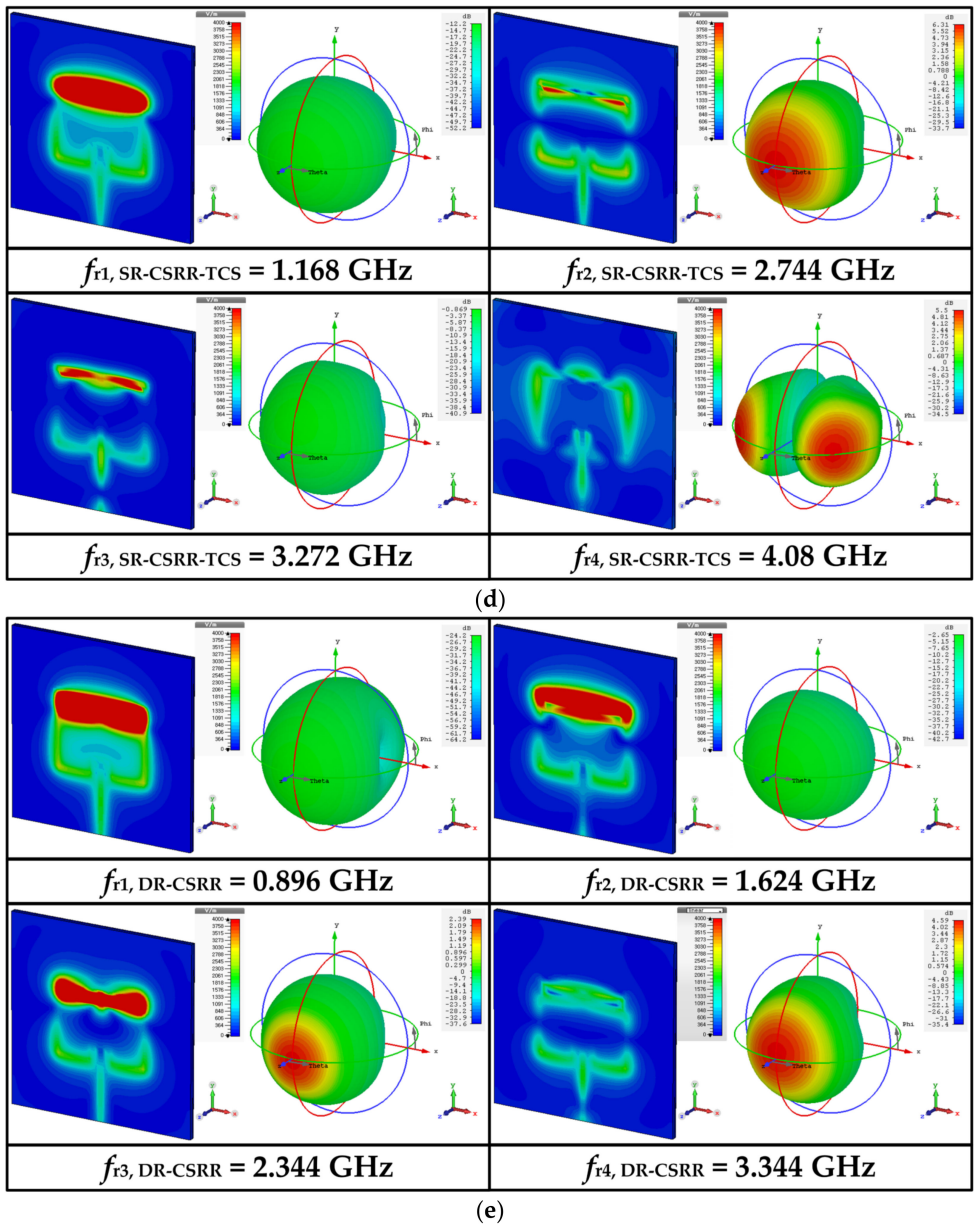
Simulated electric field distributions and radiation patterns of the reference rectangular and four slot-loaded MPAs at the first six resonant frequencies: (**a**) reference rectangular MPA, (**b**) RS-loaded MPA, (**c**) SR-CSRR-BCS slot-loaded MPA, (**d**) SR-CSRR-TCS slot-loaded MPA, and (**e**) DR-CSRR slot-loaded MPA.

Figure 1 shows the geometries of the rectangular MPAs loaded with four different types of slots near the upper radiating edge of the patch. Four different slots considered for comparison are a rectangular slot (RS), an SR-CSRR with a bottom-edge center split (SR-CSRR-BCS) slot, an SR-CSRR with a top-edge center split (SR-CSRR-TCS) slot, and a double-ring CSRR (DR-CSRR) slot. A reference inset-fed rectangular MPA without a slot was designed to have the first resonant frequency at 2.5 GHz on an RF-35 substrate (*ε*_r_ = 3.5, tan *δ* = 0.0018, *h* = 0.76 mm). This will help to differentiate the modes of the MPAs with various slots. The dimensions of the rectangular patch were calculated by using the equations from [36], and the calculated width and length of the rectangular patch were *W* = 40.0 mm and *L* = 31.9 mm, respectively. Table 1 lists all the design parameters. The simulated input reflection coefficient (S_11_) characteristics of the reference MPA under unloaded conditions within 8 GHz are presented in Figure 2, along with those of the MPAs loaded with four different types of slots. The first six resonant frequencies of the reference MPA appeared at *f*_r1,reference_ = 2.5 GHz (Transverse Magnetic (TM)_010_ mode), *f*_r2,reference_ = 4.046 GHz (TM_200_ mode), *f*_r3,reference_ = 4.776 GHz (TM_020_ mode), *f*_r4,reference_ = 5.148 GHz (TM_210_ mode), *f*_r5,reference_ = 6.452 GHz (TM_220_ mode), and *f*_r6,reference_ = 7.238 GHz (TM_030_ mode). The first and lowest resonant frequency is the TM_010_ mode, and other resonant frequencies can be considered higher-order modes. The resonant modes of the reference MPA excited by an inset-fed microstrip transmission line were analyzed using the electric field distributions and radiation patterns at the resonant frequencies, as shown in Figure 3a. In addition, the theoretical frequencies of the resonant modes when the rectangular MPA is considered a cavity can be expressed using the following equation [37].

(1) $$f_{\mathrm{mnp}}=\frac{c}{2\sqrt{\varepsilon_{\mathrm{reff}}}}\sqrt{{\left(\frac{m}{W}\right)}^{2}+{\left(\frac{n}{L_{\mathrm{eff}}}\right)}^{2}+{\left(\frac{p}{h}\right)}^{2}}$$

(2) $$\varepsilon_{\mathrm{reff}}=\frac{\left(\varepsilon_{r}+1\right)}{2}+\frac{\left(\varepsilon_{r}-1\right)}{2\sqrt{1+\frac{\left(12h\right)}{W}}}$$

(3) $$\Delta L=\frac{\left(0.412h\right)\left(\varepsilon_{\mathrm{reff}}+3\right)\left(\frac{W}{h}+3\right)}{\left(\varepsilon_{\mathrm{reff}}-0.258\right)\left(\frac{W}{h}+0.8\right)}$$

(4) $$L_{\mathrm{eff}}=L+2\Delta L$$

where: *f*_mnp_ is the resonant frequency of TM_mnp_ mode; *c* is the speed of light; *ε*_r_ is the relative permittivity of the substrate; *ε*_reff_ is the effective relative permittivity; Δ*L* is the extension of the patch length due to fringing effects; *L*_eff_ is the effective length of the patch considering Δ*L*; *m* is the integer mode number along the patch width direction (+*x* axis); *n* is the integer mode number along the patch length direction (+*y* axis); and *p* is the integer mode number along the substrate height direction (+*z* axis).

The calculated resonant frequencies appeared at *f*_TM010_ = 2.5 GHz, *f*_TM200_ = 4.081 GHz, *f*_TM210_ = 4.785 GHz, *f*_TM020_ = 5.0 GHz, *f*_TM220_ = 6.45 GHz, and *f*_TM030_ = 7.5 GHz. Although the order and values of the resonant frequencies for the TM_210_ and TM_020_ modes were changed, the simulated resonant frequencies agree well with the theoretical resonant frequencies of the patch cavity. In addition, the mode number in the resonance mode equals the number of nulls in the electric field distribution in each direction.

**Figure 4:**
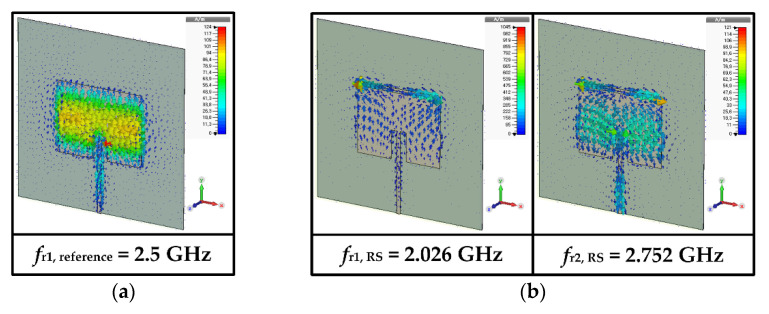

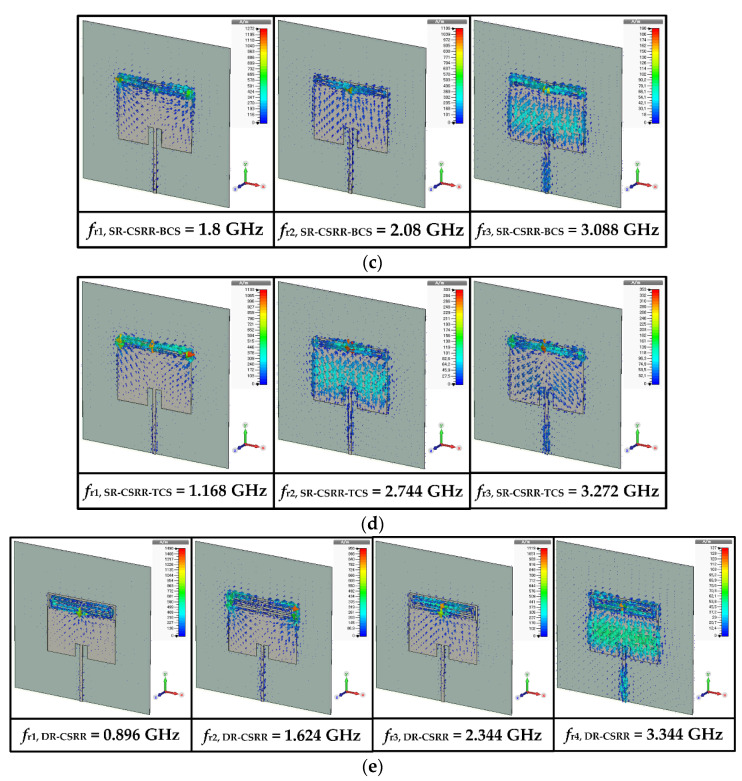
Simulated surface current distributions of the reference and four slot-loaded MPAs at the resonant frequencies with a broadside direction (+*z* axis) radiation patterns: (**a**) the reference MPA, (**b**) the RS-loaded MPA, (**c**) the SR-CSRR-BCS slot-loaded MPA, (**d**) the SR-CSRR-TCS slot-loaded MPA, and (**e**) the DR-CSRR slot-loaded MPA.

Figure 3b shows the simulated electric field distributions and radiation patterns at the resonant frequencies for the RS-loaded MPA. The first four resonant frequencies of the RS-loaded MPA were at *f*_r1,RS_ = 2.026 GHz, *f*_r2,RS_ = 2.752 GHz, *f*_r3,RS_ = 4.118 GHz, and *f*_r4,RS_ = 5.136 GHz. *f*_r1,RS_ is the TM_010_ mode of the RS-loaded patch, whereas *f*_r2,RS_ is TM_020_ mode. Figure 4b shows the surface current distributions of *f*_r1,RS_ and *f*_r2,RS_. The surface current distribution at *f*_r1,RS_ is similar to that of the TM_010_ mode for the reference MPA, as shown in Figure 4a, and the frequency location is shifted toward a lower frequency because of the loading effect of the RS. For *f*_r2,RS_, the surface currents circulate around the RS, and a null exists near the RS [38]. This perturbs the current distribution of the original TM_020_ mode with two peaks, and the currents of the perturbed TM_020_ mode become similar to those of the TM_010_ mode with a single peak, as shown in Figure 4b. Therefore, the radiation pattern of the perturbed TM_020_ mode becomes similar to that of the TM_010_ mode, which is a broadside direction (+*z* axis) radiation pattern. Other remaining resonant frequencies are similar to the higher-order modes of the reference MPA. *f*_r3,RS_ is TM_200_ mode, whereas *f*_r4,RS_ is TM_210_ mode.

Figure 3c presents the electric field distributions and radiation patterns at the resonant frequencies for the SR-CSRR-BCS slot-loaded MPA. The first four resonant frequencies of the SR-CSRR-BCS slot-loaded MPA occurred at *f*_r1,SR-CSRR-BCS_ = 1.8 GHz, *f*_r2,SR-CSRR-BCS_ = 2.08 GHz, *f*_r3,SR-CSRR-BCS_ = 3.088 GHz, and *f*_r4,SR-CSRR-BCS_ = 4.056 GHz. In this case, the first three resonant frequencies show a broadside direction radiation pattern. *f*_r1,SR-CSRR-BCS_ is the TM_010_ mode loaded with the part of the SR-CSRR-BCS slot adjacent to the edges of the patch, whereas *f*_r2,SR-CSRR-BCS_ is the TM_010_ mode loaded with the bent part of the SR-CSRR-BCS slot. *f*_r3,SR-CSRR-BCS_ is the perturbed TM_020_ mode of the SR-CSRR-BCS slot-loaded patch. This can be seen from the surface current distributions at the first three resonant frequencies of the SR-CSRR-BCS slot-loaded MPA in Figure 4c. The frequency location of the first resonant frequency, *f*_r1,SR-CSRR-BCS_, is lower than that of the first resonant frequency, *f*_r1,RS_, for the RS-loaded MPA, because of the increased slot length of the SR-CSRR-BCS. The remaining *f*_r4,SR-CSRR-BCS_ is in TM_200_ mode.

Figure 3d shows the electric field distributions and radiation patterns at the resonant frequencies of the SR-CSRR-TCS slot-loaded MPA. The first four resonant frequencies of the SR-CSRR-TCS slot-loaded MPA were at *f*_r1,SR-CSRR-TCS_ = 1.168 GHz, *f*_r2,SR-CSRR-TCS_ = 2.744 GHz, *f*_r3,SR-CSRR-TCS_ = 3.272 GHz, and *f*_r4,SR-CSRR-TCS_ = 4.08 GHz. The SR-CSRR-TCS slot-loaded MPA also has the first three resonant frequencies with a broadside direction radiation pattern. These are similar to the SR-CSRR-BCS slot-loaded MPA, but the frequency locations are different. *f*_r1,SR-CSRR-TCS_ is the TM_010_ mode of the SR-CSRR-TCS slot-loaded patch fully loaded with the SR-CSRR-TCS slot, whereas *f*_r2,SR-CSRR-TCS_ is the perturbed TM_020_ mode of the SR-CSRR-TCS-loaded patch. *f*_r3,SR-CSRR-TCS_ is the perturbed TM_030_ mode of the SR-CSRR-TCS slot-loaded patch. Figure 4d shows the surface current distributions at the first three resonant frequencies of the SR-CSRR-TCS slot-loaded MPA. The frequency location of the first resonant frequency, *f*_r1,SR-CSRR-TCS_, is much lower than that of the first resonant frequency, *f*_r1,SR-CSRR-BCS_, of the SR-CSRR-BCS slot-loaded MPA, because of the increased current path with different split locations. The remaining *f*_r4,SR-CSRR-TCS_ is in TM_200_ mode.

Figure 3e shows the electric field distributions and radiation patterns at the resonant frequencies for the DR-CSRR slot-loaded MPA. The first four resonant frequencies of the DR-CSRR slot-loaded MPA appeared at *f*_r1,DR-CSRR_ = 0.896 GHz, *f*_r2,DR-CSRR_ = 1.624 GHz, *f*_r3,DR-CSRR_ = 2.344 GHz, and *f*_r4,DR-CSRR_ = 3.344 GHz. In this case, the first four resonant frequencies show a broadside direction radiation pattern. The whole DR-CSRR slot-loaded patch acts as a folded radiator at *f*_r1,DR-CSRR_, whereas *f*_r2,DR-CSRR_ is the TM_010_ mode of the DR-CSRR slot-loaded patch loaded with the outer ring part of the DR-CSRR slot adjacent to the edges of the patch. The first resonant frequency (*f*_r1,DR-CSRR_) of the DR-CSRR slot-loaded MPA is the lowest among the four slot-loaded MPAs. *f*_r3,DR-CSRR_ is the TM_010_ mode of the DR-CSRR slot-loaded patch loaded with the inner part of the DR-CSRR, whereas *f*_r4,DR-CSRR_ is the perturbed TM_030_ mode of the DR-CSRR slot-loaded patch. Figure 4e shows the surface current distributions at the first four resonant frequencies of the DR-CSRR slot-loaded MPA.

## 3. Sensitivity Comparison of Reference Rectangular and Four Different Slot-Loaded MPAs for First Four Resonant Frequencies

Based on the analysis results of the resonant frequencies for the reference rectangular and the four slot-loaded MPAs, the sensitivities of the first four resonant frequencies were compared by measuring the shifts in the resonant frequencies of the input reflection coefficient characteristics when the material under test (MUT) was placed above the patch as a superstrate with varying relative permittivity.

**Figure 5:**
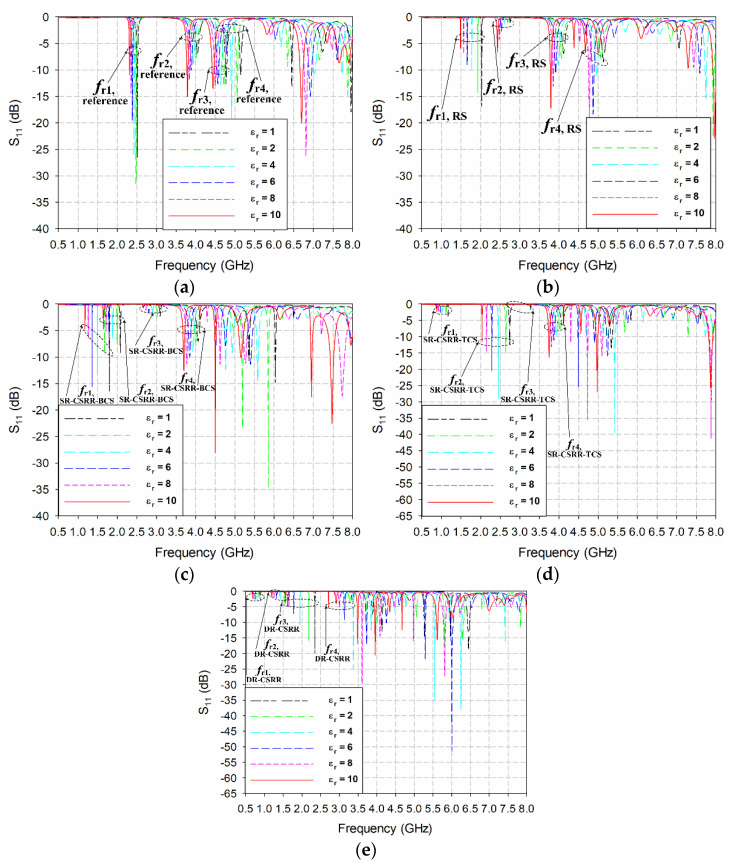
Input reflection coefficient responses of the reference and four slot-loaded MPAs for varying the relative permittivity of the MUT superstrate from 1 to 10: (**a**) the reference rectangular MPA, (**b**) the RS-loaded MPA, (**c**) the SR-CSRR-BCS slot-loaded MPA, (**d**) the SR-CSRR-TCS slot-loaded MPA, and (**e**) the DR-CSRR slot-loaded MPA.

**Figure 6:**
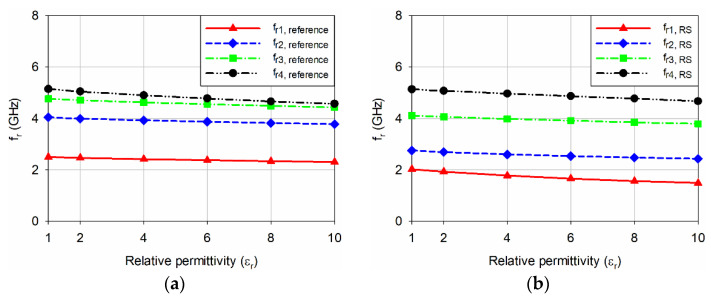

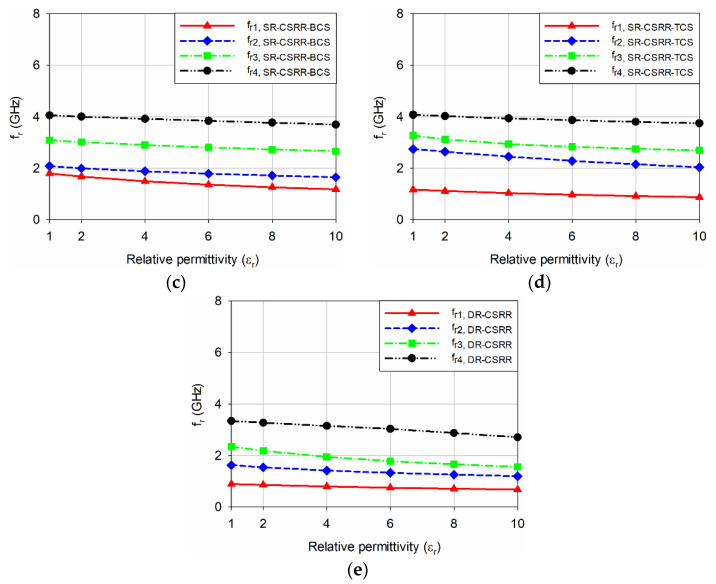
Comparison of the first four resonant frequencies for the reference and four slot-loaded MPAs when varying the relative permittivity of the MUT superstrate from 1 to 10: (**a**) reference rectangular MPA, (**b**) RS-loaded MPA, (**c**) SR-CSRR-BCS slot-loaded MPA, (**d**) SR-CSRR-TCS slot-loaded MPA, and (**e**) DR-CSRR slot-loaded MPA.

**Figure 7:**
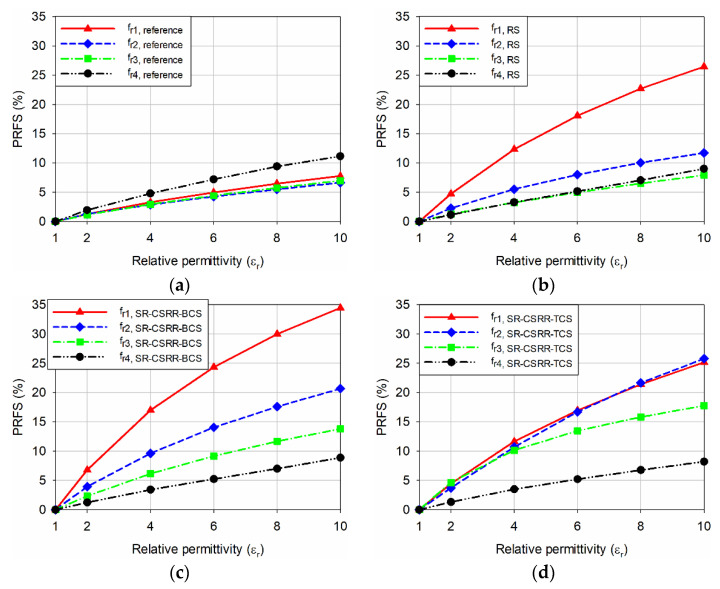

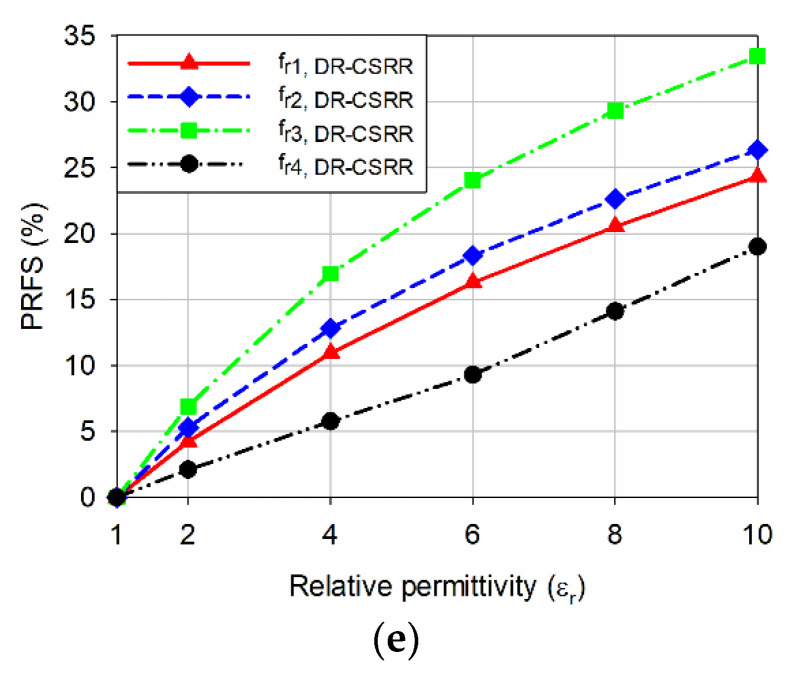
PRFS of the first four resonant frequencies for the reference and four slot-loaded MPAs when varying the relative permittivity of the MUT superstrate from 1 to 10: (**a**) the reference rectangular MPA, (**b**) the RS-loaded MPA, (**c**) the SR-CSRR-BCS slot-loaded MPA, (**d**) the SR-CSRR-TCS slot-loaded MPA, and (**e**) the DR-CSRR slot-loaded MPA.

Figure 5 shows the input reflection coefficient responses of the reference and four slot-loaded MPAs when varying the relative permittivity of the MUT superstrate from 1 to 10 with a loss tangent = 0. The thickness of the MUT superstrate was chosen at 1.6 mm [34], and the dimensions of the MUT were the same as the ground plane. Figure 6 shows the first four resonant frequencies of the reference and four slot-loaded MPAs, and Table 2 lists their values. Because the values of the resonant frequencies are all different and it is difficult to compare the sensitivity for each resonant frequency directly using the frequency shift itself, the percentage relative frequency shift (PRFS) of the resonant frequencies was defined using Equation (5) to compare the relative permittivity sensitivity of the first four resonant frequencies for the reference and four slot-loaded MPAs. Figure 7 shows the PRFS characteristics of the first four resonant frequencies for the reference and four slot-loaded MPAs.

(5) $$\mathrm{PRFS}\;(\%)=\frac{\Delta f_{r}}{f_{r}}\times 100\;(\%)=\frac{f_{r,\mathrm{loaded}}{-f}_{r,\mathrm{unloaded}(\mathrm{air})}}{f_{r,\mathrm{unloaded}(\mathrm{air})}}\times 100\;(\%)$$

where Δ*f*_r_ is the shift in the resonant frequencies, *f*_r,unloaded(air)_ is the resonant frequencies for unloaded conditions (air), and *f*_r,loaded_ is the resonant frequencies for loaded conditions with MUTs.

The PRFS of the first four resonant frequencies for the reference MPA was smaller than 11.2% when the relative permittivity of the MUT varied from 1 to 10, and that of the fourth resonant frequency, *f*_r4,reference_, was the largest (Figure 7a). When the RS was loaded on the patch of the reference MPA, the PRFS of the first resonant frequency, *f*_r1,RS_, was increased to 26.5%, with the largest enhancement. On the other hand, the PRFS of the remaining resonant frequencies was less than 11.7%. For the SR-CSRR-BCS slot-loaded MPA, the PRFS of *f*_r1,SR-CSRR-BCS_ was increased to 34.4%, whereas PRFS of *f*_r2,SR-CSRR-BCS_, *f*_r3,SR-CSRR-BCS_, and *f*_r4,SR-CSRR-BCS_ were 20.7%, 13.8%, and 8.9%, respectively. Therefore, the first resonant frequency, *f*_r1,SR-CSRR-BCS_, has the largest PRFS among the four, and its sensitivity enhancement was better than *f*_r1,RS_. When the SR-CSRR-TCS slot with the split position close to the radiating edge was loaded on the patch, the PRFS of *f*_r1,SR-CSRR-TCS_, *f*_r2,SR-CSRR-TCS_, *f*_r3,SR-CSRR-TCS_, and *f*_r4,SR-CSRR-TCS_ were 25.2%, 25.8%, 17.8%, and 8.2%, respectively. PRFS of *f*_r1,SR-CSRR-TCS_, and *f*_r2,SR-CSRR-TCS_ were similar, but they are smaller than that of *f*_r1,SR-CSRR-BCS_. Although the frequency location of *f*_r1,SR-CSRR-TCS_ was much lower than that of *f*_r1,SR-CSRR-BCS_, the sensitivity enhancement is not good compared to *f*_r1,SR-CSRR-BCS_. Finally, for the DR-CSRR slot-loaded MPA, PRFS of *f*_r1,DR-CSRR_, *f*_r2,DR-CSRR_, *f*_r3,DR-CSRR_, and *f*_r4,DR-CSRR_ were 24.33%, 26.4%, 33.5%, and 19.0%, respectively. In this case, the PRFS of the third resonant frequency, *f*_r3,DR-CSRR_, was the largest, but it was smaller than that of *f*_r1,SR-CSRR-BCS_. Note that the frequency location of *f*_r1,DR-CSRR_ is lower than that of *f*_r1,SR-CSRR-TCS_ and *f*_r1,SR-CSRR-BCS_, but the sensitivity enhancement was no better. Therefore, the PRFS of the first resonant frequency, *f*_r1,SR-CSRR-TCS_, for the SR-CSRR-BCS slot-loaded MPA is the largest among the first four resonant frequencies of the reference and four slot-loaded MPAs.

**Figure 8:**
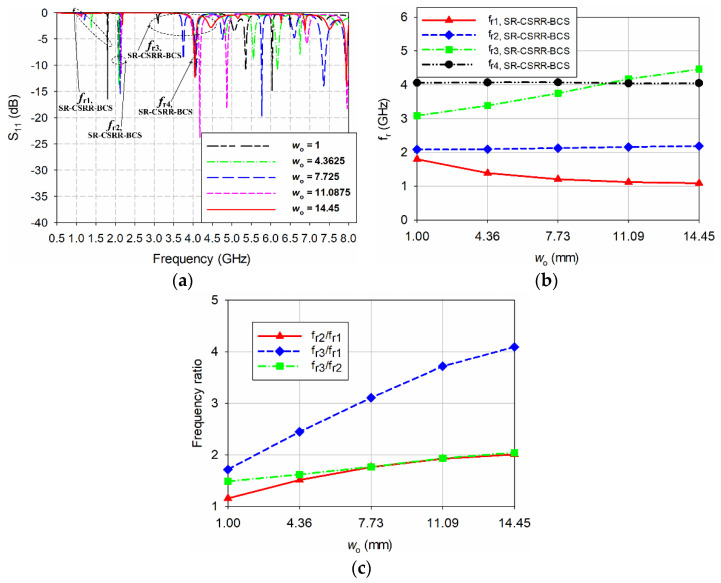
Effect of varying the offset *w*_o_ of the SR-CSRR-BCS slot from the radiating edge (*w*_o_ = 1 mm) toward the patch center (*w*_o_ = 14.45 mm) on the resonant frequencies of the SR-CSRR-BCS slot-loaded MPA: (**a**) input reflection coefficient characteristic, (**b**) first four resonant frequencies, and (**c**) frequency ratio between the first three resonant frequencies.

The effect of varying the position of the SR-CSRR-BCS slot in the patch on the resonant frequencies was next investigated, as shown in Figure 8. In this case, the offset *w*_o_ from the radiating edge was changed from 1 mm (near the radiating edge) to 14.45 mm (center of the patch) with a step of 3.3625 mm. When the offset *w*_o_ was increased from 1 mm to 14.45 mm so that the position of the SR-CSRR-BCS slot moved from near the radiating edge to the center of the patch, the first resonant frequency *f*_r1,SR-CSRR-BCS_ decreased quadratically from 1.8 GHz to 1.09 GHz. In contrast, the second resonant frequency *f*_r2,SR-CSRR-BCS_ increased slightly from 2.08 GHz to 2.186 GHz. The third resonant frequency *f*_r3,SR-CSRR-BCS_ increased almost linearly from 3.088 GHz to 4.46 GHz, whereas the fourth resonant frequency *f*_r4,SR-CSRR-BCS_ remained relatively constant between 4.046 GHz and 4.07 GHz. The frequency ratio between the first and second resonant frequencies (*f*_r2,SR-CSRR-BCS_/*f*_r1,SR-CSRR-BCS_) increased quadratically from 1.16 to 2.00, and the frequency ratio between the first and third resonant frequencies (*f*_r3,SR-CSRR-BCS_/*f*_r1,SR-CSRR-BCS_) increased quadratically from 1.72 to 4.09. The frequency ratio between the second and third resonant frequencies (*f*_r3,SR-CSRR-BCS_/*f*_r2,SR-CSRR-BCS_) increased linearly from 1.48 to 2.04. Therefore, if the proposed SR-CSRR-BCS slot-loaded MPA is used for a multiband antenna, the frequency ratio between the three resonant frequencies can be adjusted by changing the offset of the SR-CSRR-BCS slot from the radiating edge. In addition, impedance matching at the first resonant frequency deteriorated as the position of the slot moved toward the patch center.

**Figure 9:**
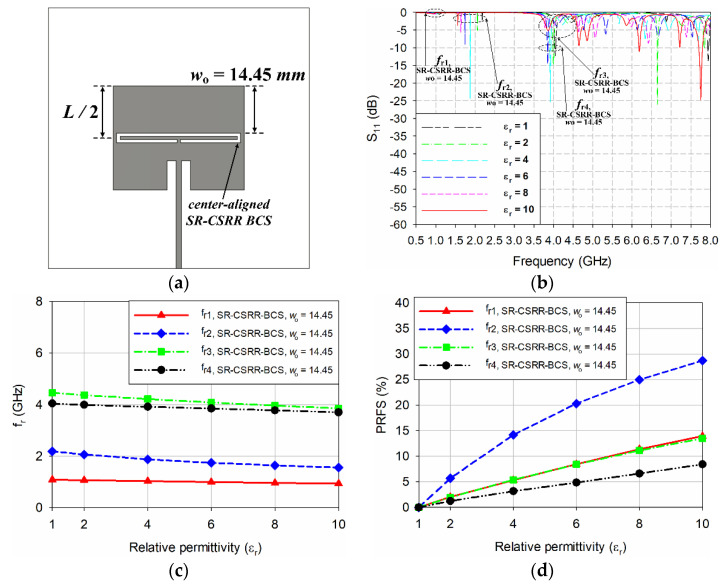
Characteristics of the center-aligned SR-CSRR-BCS slot-loaded MPA (*w*_o_ = 14.45 mm): (**a**) antenna geometry, (**b**) input reflection coefficient characteristic, (**c**) values of the first four resonant frequencies, and (**d**) PRFS of the first four resonant frequencies.

Finally, this study compared the input reflection coefficient characteristics, the values of the first four resonant frequencies, and the PRFS of the SR-CSRR-BCS slot-loaded MPA, when the SR-CSRR-BCS slot moved to the center of the patch (*w*_o_ = 14.45 mm). Figure 9a shows the geometry of the center-aligned SR-CSRR-BCS slot-loaded MPA. The first four resonant frequencies of the center-aligned SR-CSRR-BCS slot-loaded MPA appeared at *f*_r1,SR-CSRR-BCS,*w*o=14_._45_ = 1.09 GHz, *f*_r2,SR-CSRR-BCS,*w*o=14_._45_ = 2.186 GHz, *f*_r3,SR-CSRR-BCS,*w*o=14_._45_ = 4.46 GHz, and *f*_r4,SR-CSRR-BCS,*w*o=14_._45_ = 4.046 GHz. The PRFS of *f*_r1,SR-CSRR-BCS,*w*o=14_._45_, *f*_r2,SR-CSRR-BCS,*w*o=14_._45_, *f*_r3,SR-CSRR-BCS,*w*o=14_._45_, and *f*_r4,SR-CSRR-BCS,*w*o=14_._45_ were 13.9%, 28.7%, 13.5%, and 8.4%, respectively, when the relative permittivity of the MUT superstrate varied from 1 to 10. The PRFS of the second resonant frequency, *f*_r2,SR-CSRR-BCS,*w*o=14_._45_, was the largest among the four, but it was no better than that of the first resonant frequency for the radiating edge-aligned SR-CSRR-BCS slot-loaded MPA in Figure 1b. Therefore, the center-aligned SR-CSRR-BCS slot-loaded MPA might be useful for achieving a lower first resonant frequency with large size reduction and a large frequency ratio with the second and third resonant frequencies. However, its sensitivity to the relative permittivity was no better compared to the radiating edge-aligned SR-CSRR-BCS slot-loaded MPA.

## 4. Design of Radiating Edge-Aligned SR-CSRR-BCS Slot-Loaded MPA for Sensing Microliter-Volume Liquid Chemicals with High Relative Permittivity and High Loss Tangent

Because the sensitivity of the first resonant frequency of the SR-CSRR-BCS slot-loaded MPA is the highest among the first four resonant frequencies of the reference and four slot-loaded MPAs, the SR-CSRR-BCS slot-loaded MPA was selected for sensing microliter-volume liquid chemicals with high relative permittivity and high loss tangent.

**Figure 10:**
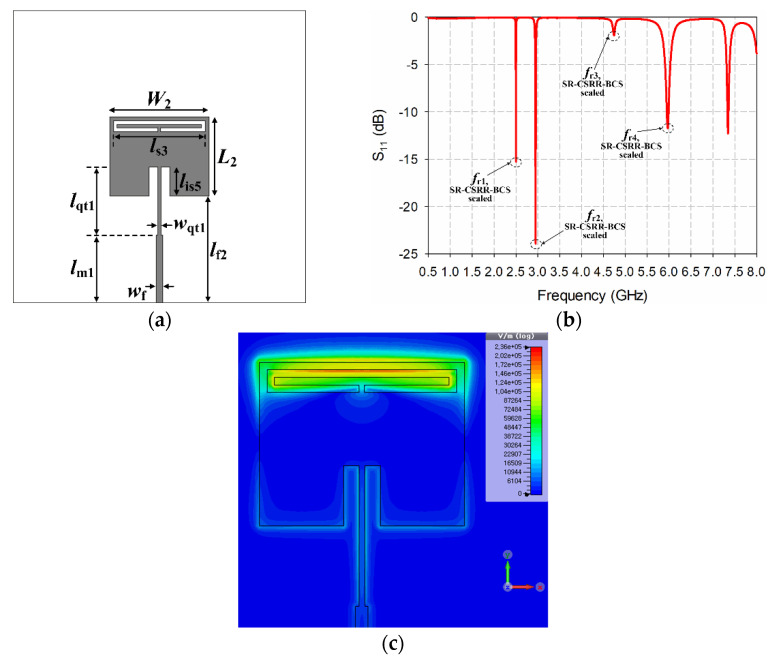
Scaled radiating edge-aligned SR-CSRR-BCS slot-loaded MPA with the first resonant frequency at 2.5 GHz: (**a**) antenna geometry, (**b**) input reflection coefficient characteristic, and (**c**) electric field distribution at 2.5 GHz.

First, the width and length of the patch and the length of the SR-CSRR-BCS slot were scaled down to shift the first resonant frequency at 2.5 GHz without the MUT, as shown in Figure 10a. The scaled dimensions for the width and length of the patch and the length of the SR-CSRR-BCS slot were *W*_2_ = 27.2 mm, *L*_2_ = 21.7 mm, and *l*_s3_ = 25.2 mm, respectively. The patch dimensions were reduced by 32% compared to the reference MPA. A quarter-wavelength transformer with *w*_qt1_ = 0.84 mm and *l*_qt1_ = 18.7 mm was applied to the microstrip feed line to improve input impedance matching. The other parameters were *l*_m1_ = 18.5 mm, *l*_is5_ = 8 mm, and *l*_f2_ = 29.2 mm. The first four resonant frequencies of the scaled-down radiating edge-aligned SR-CSRR-BCS slot-loaded MPA were *f*_r1,SR-CSRR-BCS,scaled_ = 2.5 GHz, *f*_r2,SR-CSRR-BCS,scaled_ = 2.95 GHz, *f*_r3,SR-CSRR-BCS,scaled_ = 4.742 GHz, and *f*_r4,SR-CSRR-BCS,scaled_ = 5.97 GHz, as shown in Figure 10b. Figure 10c shows the electric field distribution at the first resonant frequency. The electric field distribution reached a maximum near the center of the top-edge for the SR-CSRR-BCS slot. Therefore, the center of the top-edge for the SR-CSRR-BCS slot was chosen for the location of the liquid container.

**Figure 11:**
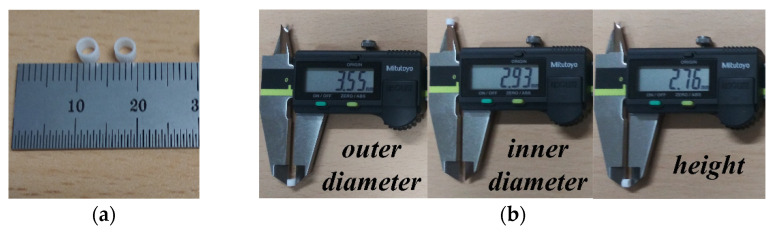
Fabricated liquid container: (**a**) photograph and (**b**) measured dimensions.

A hollow cylindrical liquid container made of acryl (*ε*_r_ = 2.56) was designed and fabricated using a laser cutting system (VLS 3.50 model, Universal Laser Systems, Scottsdale, AZ, USA), as shown in Figure 11. The dimensions of the fabricated liquid container were measured using a digital Vernier caliper (500-181-30, Mitutoyo Co., Kawasaki, Kanagwa, Japan). The measured outer diameter, inner diameter, and height of the fabricated liquid container were 3.55 mm, 2.93 mm, and 2.76 mm, respectively. Therefore, the inner volume of the fabricated liquid container is approximately 18.6 μL.

**Figure 12:**
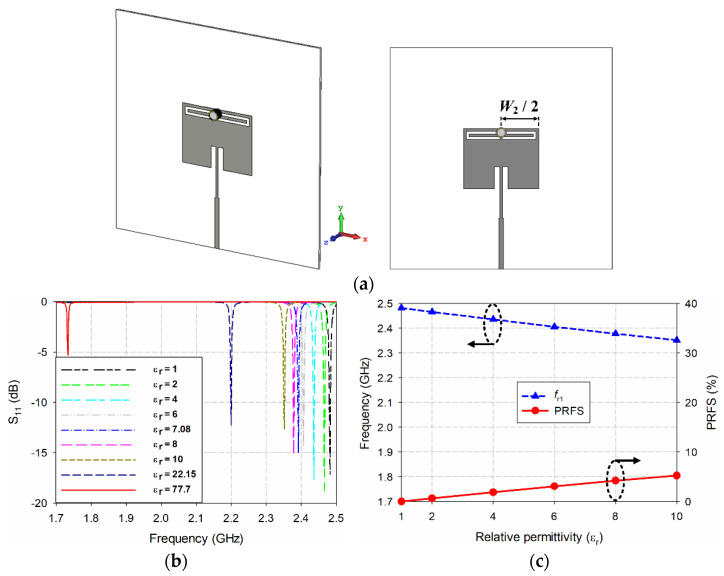

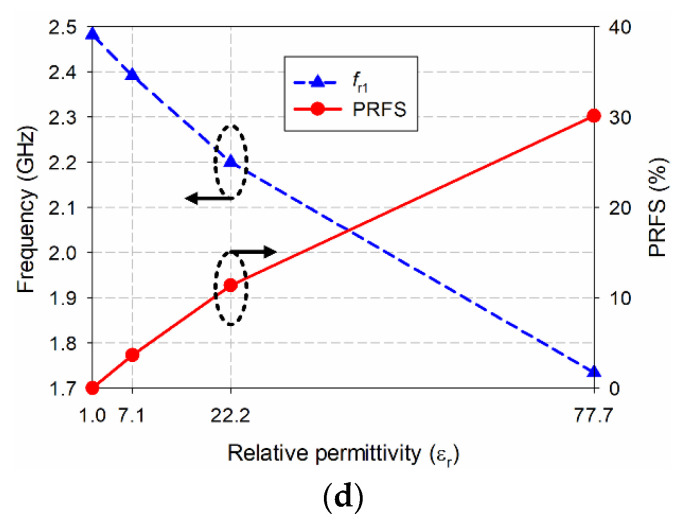
Effects of varying the relative permittivity of the MUT inside the liquid container on the sensitivity of the scaled radiating edge-aligned SR-CSRR-BCS slot-loaded MPA with the liquid container at the center of the top-edge: (**a**) geometry, (**b**) input reflection coefficient characteristics, (**c**) values and PRFS of the first resonant frequency when the MUT’s relative permittivity varied from 1 to 10, and (**d**) the values and PRFS of the first resonant frequency for the MUT with the relative permittivity of ethanol, methanol, and water.

This study next examined the effects of varying the relative permittivity of the MUT inside the liquid container on the sensitivity of the first resonant frequency for the scaled radiating edge-aligned SR-CSRR-BCS slot-loaded MPA when the liquid container was placed at the center of the top edge (Figure 12a). The relative permittivities of the MUT inside the liquid container for the simulation were 1, 2, 4, 6, 8, and 10, which were used for the MUT superstrate. In addition, the relative permittivities of ethanol, methanol, and water, which were selected for testing polar liquids to cover a broad range of high relative permittivity and high loss tangents, were also considered. Table 3 lists the relative permittivity and loss tangent values of ethanol, methanol, and water at 2.5 GHz and 25 °C [39,40]. Figure 12 presents the simulation results of the input reflection coefficient characteristics, the values of the first resonant frequency, and PRFS of the first resonant frequency of the scaled SR-CS-BCS slot-loaded MPA with the liquid container at the center of the top edge.

When the relative permittivity of the MUT inside the cylindrical liquid container varied from 1 to 10, the first resonant frequency decreased almost linearly from 2.482 GHz to 2.352 GHz. The PRFS of the first resonant frequency increased to 5.24%, which is much smaller than the case when the MUT superstrate with a thickness of 1.6 mm was used. This might be caused by the smaller dimensions and sensing area of the MUT inside the liquid container. The volume of the MUT superstrate is approximately 10,240 μL, which is approximately 550 times larger than that of the MUT inside the liquid container. When the relative permittivities of ethanol (*ε*_r_ = 7.08), methanol (*ε*_r_ = 22.15), and water (*ε*_r_ = 77.7) were applied, the first resonant frequency decreased quadratically to 2.392 GHz, 2.2 GHz, and 1.734 GHz, respectively; its PRFS increased to 3.63%, 11.36%, and 30.14%, respectively.

**Figure 13:**
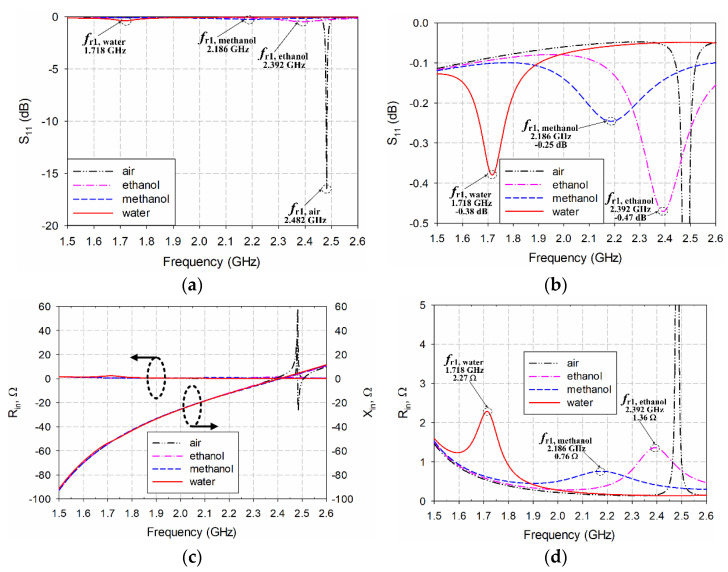
Responses of the scaled radiating edge-aligned SR-CSRR-BCS slot-loaded MPA with the liquid container at the center of the top-edge when loss tangent of ethanol, methanol, and water was applied for the MUT: (**a**) input reflection coefficient with full magnitude range, (**b**) input reflection coefficient with zoomed-in magnitude range, (**c**) input impedance, and (**d**) zoomed-in input resistance.

The effects of applying the loss tangents of ethanol, methanol, and water on the performance characteristics of the scaled radiating edge-aligned SR-CSRR-BCS slot-loaded MPA with the liquid container were studied. Figure 13 presents the input reflection coefficient and impedance characteristics of the first resonant frequency for the scaled radiating edge-aligned SR-CSRR-BCS slot-loaded MPA with the liquid container when the relative permittivity and loss tangents of ethanol, methanol, and water were used to simulate the MUT inside the liquid container. Figure 13 shows that the magnitude of the input reflection coefficient at the first resonant frequency when ethanol, methanol, and water became close to 0 dB, indicating a large reflection because of impedance mismatching between the 50 ohm (Ω) feedline and the patch, caused by very small input resistance at the first resonant frequency. In addition, the input reactance value at the first resonant frequency became a large negative number (capacitive). Small input resistance and negative large input reactance were caused by high loss tangents of ethanol, methanol, and water. The magnitudes of the input reflection coefficients for liquid MUTs with ethanol, methanol, and water were −0.47 dB, −0.25 dB, and −0.38 dB, respectively, whereas the input resistance values were 1.36 ohms, 0.76 ohms, and 2.27 ohms, respectively.

**Figure 14:**
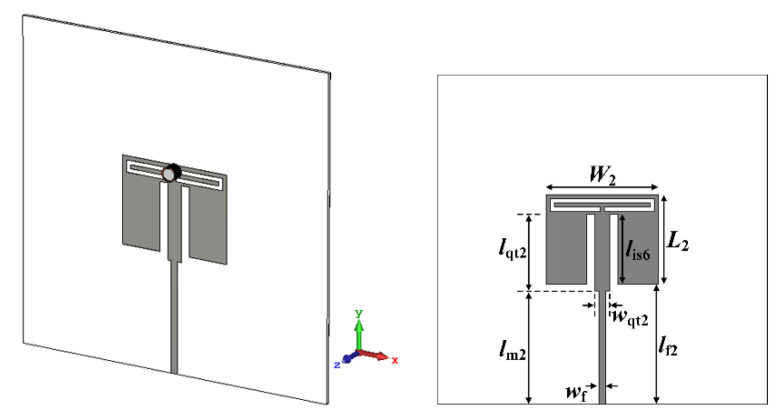
Geometry of the quarter-wave transformer-added scaled radiating edge-aligned SR-CSRR-BCS slot-loaded MPA with the liquid container for liquid MUTs with high relative permittivity and high loss tangent.

A quarter-wavelength impedance transformer was designed to improve impedance mismatching caused by high loss tangents of MUTs. It was appended between the 50 Ω microstrip feedline and the patch with low resistance, as shown in Figure 14. The dimensions of the quarter-wavelength transformer were *l*_qt2_ = 18.7 mm and *w*_qt2_ = 3.66 mm, and other parameters are *l*_m2_ = 27.5 mm and *l*_is6_ = 17 mm. Note that the width of the quarter-wavelength transformer is wider than the 50 Ω feedline for impedance matching between 50 Ω of the feedline and a few ohms of the patch. Although the input reflection coefficient for air without the MUT was increased to −1.18 dB, the input reflection coefficients for ethanol, methanol, and water were decreased to −30.96 dB, −17.9 dB, and −21.05 dB, respectively, with enhanced impedance matching. In addition, the first resonant frequency for air moved toward the high frequency to 2.696 GHz, whereas that for ethanol was also slightly increased to 2.42 GHz. On the other hand, the first resonant frequencies for methanol and water were decreased to 2.412 GHz and 1.63 GHz. Therefore, the PRFS of the first resonant frequencies for ethanol, methanol, and water were increased to 10.24%, 20.55%, and 39.54%, respectively, compared to the zero loss tangent cases in Figure 12.

**Figure 15:**
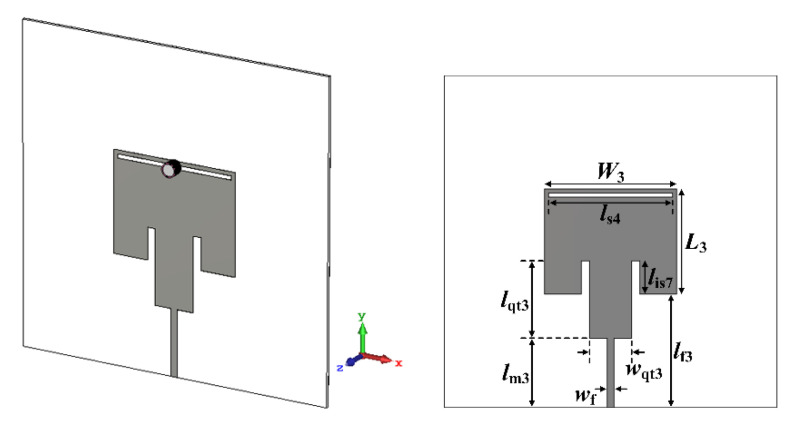
Geometry of the quarter-wave transformer-added scaled RS-loaded MPA with the liquid container for liquid MUTs with high relative permittivity and high loss tangent.

Finally, the scaled radiating edge-aligned RS-loaded MPA with the liquid container considering the high loss tangent of ethanol, methanol, and water was designed for the purpose of sensitivity comparison, as shown in Figure 15. The width and length of the patch and the length of the RS, in Figure 1a, were scaled down approximately 20.4% to shift the first resonant frequency at 2.5 GHz without the MUT [34]. The scaled dimensions for the width and length of the patch and the length of the RS were *W*_3_ = 31.8 mm, *L*_3_ = 25.4 mm, and *l*_s4_ = 29.8 mm, respectively. A quarter-wavelength transformer for impedance matching of high loss tangent MUTs was also designed and appended. The dimensions of the quarter-wavelength transformer were *l*_qt3_ = 18.7 mm and *w*_qt3_ = 10 mm, and the other parameters were *l*_m3_ = 16.6 mm and *l*_is7_ = 8 mm. Similar to the case of the scaled radiating edge-aligned SR-CSRR-BCS slot-loaded MPA, impedance matching for ethanol, methanol, and water was improved, but that for air deteriorated. The input reflection coefficients for ethanol, methanol, and water were decreased to −9.63 dB, −7.06 dB, and −11.5 dB, respectively, whereas that for air was increased to −1.56 dB, as shown in Figure 18a. In this case, the first resonant frequencies for air, ethanol, methanol, and water were 2.584 GHz, 2.512 GHz, 2.33 GHz, and 1.838 GHz, respectively. The PRFS of the first resonant frequencies for ethanol, methanol, and water increased to 2.79%, 9.83%, and 28.87%, respectively. The PRFS enhancement (PRFSE) was similar to sensitivity enhancement, which can be used for a sensitivity comparison [34]. PRFSE is defined as a ratio of the PRFS for the proposed scaled radiating edge-aligned SR-CSRR-BCS slot-loaded MPA to PRFS for the scaled radiating edge-aligned RS-loaded MPA. Simulated PRFSE of the proposed scaled radiating edge-aligned SR-CSRR-BCS slot-loaded MPA, compared to the scaled radiating edge-aligned RS-loaded MPA, was calculated in Figure 18e using the PRFS in Figure 18c; PRFSE for ethanol, methanol, and water were 3.67, 2.09, and 1.37, respectively. The PRFSE for the MUT with a low relative permittivity is higher than that for the high relative permittivity MUT.

(6) $$PRFSE\;=\frac{{PRFS}_{\mathrm{SR}-\mathrm{CSRR}-\mathrm{BCS}}}{{PRFS}_{\mathrm{RS}}}$$

## 5. Liquid Chemicals Experiment Result and Discussion

**Figure 16:**
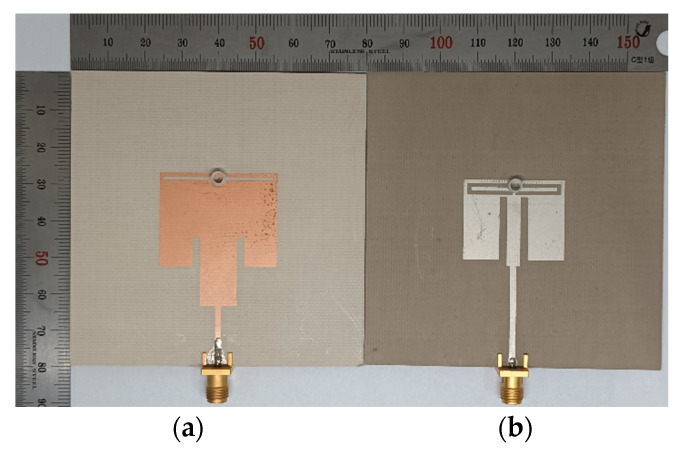
Fabricated MPAs with the liquid container: (**a**) RS-loaded MPA, and (**b**) SR-CSRR-BCS slot-loaded MPA.

Figure 16 shows the prototypes of the scaled radiating edge-aligned RS-loaded and SR-CSRR-BCS slot-loaded MPAs with impedance-matching quarter-wavelength transformers were fabricated on an 80 mm × 80 mm RF-35 substrate (*ε*_r_ = 3.5, tan *δ* = 0.0018, *h* = 0.76 mm). The fabricated cylindrical liquid containers in Figure 11 were glued using silicon onto the center of the RS or the top-edge center of the SR-CSRR-BCS slot, as shown in Figure 16.

**Figure 17:**
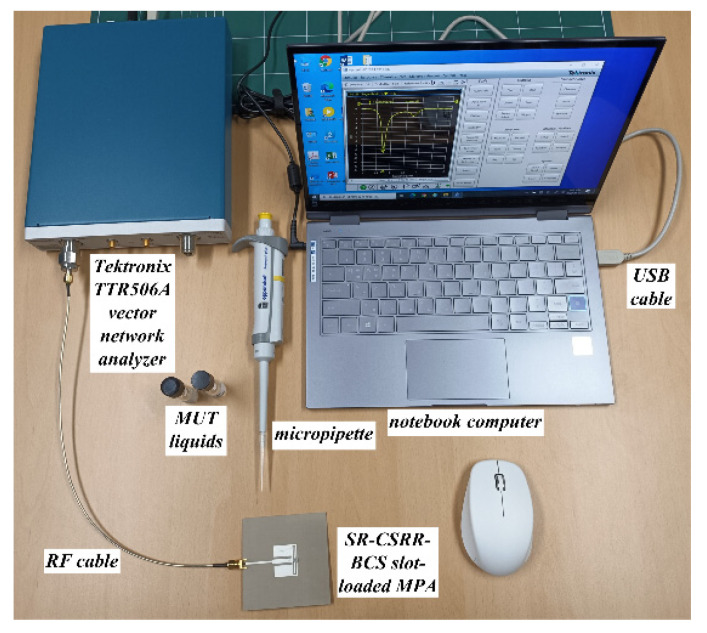
Measurement setup with a VNA and a notebook computer.

**Figure 18:**
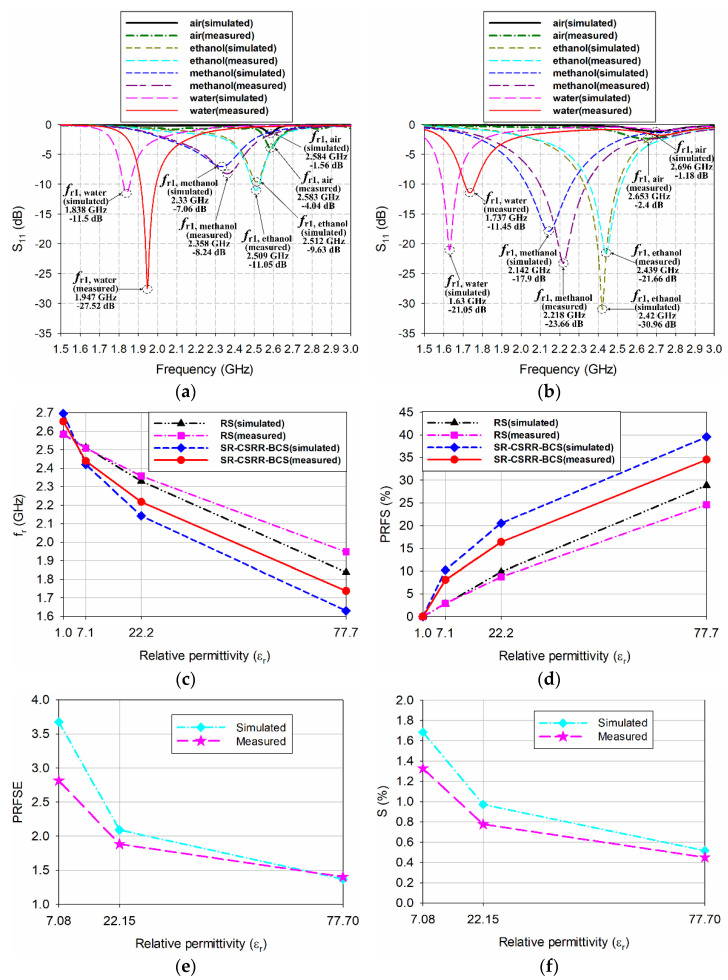
Measured results of the fabricated RS-loaded and SR-CSRR-BCS slot-loaded MPAs: (**a**) input reflection coefficient characteristics of the fabricated RS-loaded MPA, (**b**) input reflection coefficient characteristics of the fabricated SR-CSRR-BCS slot-loaded MPA, (**c**) the values of the first resonant frequency, (**d**) PRFS of the first resonant frequency, (**e**) PRFSE of the first resonant frequency for the SR-CSRR-BCS slot-loaded MPA compared to that of the RS-loaded MPA, and (**f**) sensitivity (%) of the first resonant frequency for the SR-CSRR-BCS slot-loaded MPA.

The input reflection coefficient characteristics of the fabricated MPAs were measured using a Tektronix TTR506A vector network analyzer (VNA), and Figure 17 presents a photograph of the experimental setup. Tektronix TTR506A VNA can be communicated over a universal serial bus (USB) to a notebook computer with real-time data acquisition and higher reliability. The liquid container was filled with 15 μL of ethanol, methanol, and deionized (DI) water at 25 °C using a micropipette (3120000038, Eppendorf SE, Hamburg, Germany). Figure 18 shows the measured input reflection coefficients of the scaled radiating edge-aligned RS-loaded and SR-CSRR-BCS slot-loaded MPAs with impedance-matching quarter-wavelength transformers.

For the RS-loaded MPA, the first resonant frequencies for air, ethanol, methanol, and water were 2.583 GHz, 2.509 GHz, 2.358 GHz, and 1.947 GHz, respectively. In contrast, the corresponding magnitudes of the input reflection coefficients at the first resonant frequencies were −4.04 dB, −11.05 dB, −8.24 dB, and −27.52 dB, respectively. The PRFS of the first resonant frequencies for ethanol, methanol, and water were 2.86%, 8.71%, and 24.61%, respectively.

For the SR-CSRR-BCS slot-loaded MPA, the first resonant frequencies for air, ethanol, methanol, and water were 2.653 GHz, 2.439 GHz, 2.218 GHz, and 1.737 GHz, respectively. In contrast, the corresponding magnitudes of the input reflection coefficients at the first resonant frequencies were −2.4 dB, −21.66 dB, −23.31 dB, and −11.45 dB, respectively. The PRFS of the first resonant frequencies for ethanol, methanol, and water were 8.07%, 16.4%, and 34.53%, respectively. The measured PRFSE for ethanol, methanol, and water were 2.82, 1.88, and 1.4, respectively. We can see that measured PRFSE for ethanol and methanol was lower than the simulated one, but that for water was slightly higher. The simulated and measured sensitivity (%) of the proposed SR-CSRR-BCS slot-loaded MPA were calculated using Equation (7) to quantify the sensitivity of the proposed SR-CSRR-BCS slot-loaded MPA and compare it with other microwave sensors [13].

(7) $$S\;(\%)=\frac{PRFS\;(\%)}{\Delta \varepsilon_{r}}=\frac{\Delta f_{r}\times 100\;(\%)}{f_{r,\mathrm{unloaded}(\mathrm{air})}\times \Delta \varepsilon_{r}}=\frac{\left(f_{r,\mathrm{loaded}}{-f}_{r,\mathrm{unloaded}(\mathrm{air})}\right)}{f_{r,\mathrm{unloaded}(\mathrm{air})}\times \left(\varepsilon_{r,\mathrm{loaded}}-\varepsilon_{r,\mathrm{unloaded}(\mathrm{air})}\right)}\times 100\;(\%)$$

The simulated sensitivity (%) for ethanol, methanol, and water was 1.68%, 0.97%, and 0.52%, respectively, whereas the measured sensitivity (%) was 1.33%, 0.78%, and 0.45%, respectively. The sensitivity (%) was decreased as the relative permittivity of the MUT liquid increased. The measured results are consistent with the simulated ones, with some differences. The differences might be caused by fabrication and measurement errors, including the uncertainties in the relative permittivity and loss tangent of the substrate of the MPAs and MUT liquids.

The measured sensitivity (%) of the first resonant frequency for the proposed SR-CSRR-BCS slot-loaded MPA for DI water was compared with that of other microwave liquid sensors in the literature, including antenna-based liquid sensors, as shown in Table 4. The sensitivity (%) of the proposed SR-CSRR-BCS slot-loaded MPA was higher than other antenna-based microwave sensors. It was slightly lower than that in [13], but the liquid volume in [13] was 5.3 times larger than that of the proposed SR-CSRR-BCS slot-loaded MPA.

## 6. Conclusions

This paper proposed a high-sensitivity SR-CSRR-BCS slot-loaded MPA-based microwave sensor to measure microliter-volume liquid chemicals with high relative permittivity and high loss tangent. First, the frequency locations and mode characteristics of the first four resonant frequencies of the proposed SR-CSRR-BCS slot-loaded MPA were compared with the reference MPA without the slot, three other slot-loaded MPAs with the RS, the SR-CSRR-TCS slot, and the DR-CSRR slot, using the electric field distributions, radiation patterns, and surface current distributions. The first resonant frequency of the DR-CSRR slot-loaded MPA is the lowest among the five MPAs. Next, the sensitivities of the first four resonant frequencies for the five MPAs were compared by measuring the shifts in the resonant frequencies of the input reflection coefficient characteristics, when the MUT with a thickness of 1.6 mm was placed above the patch as a superstrate with varying its relative permittivity from 1 to 10. The sensitivity of the first resonant frequency for the proposed SR-CSRR-BCS slot-loaded MPA was the highest among the first four resonant frequencies of the five MPAs, which was chosen for sensing microliter-volume liquid chemicals with high relative permittivity and high loss tangent. The effect of varying the position of the SR-CSRR-BCS slot in the patch on the resonant frequencies was also studied. A scaled SR-CSRR-BCS slot-loaded MPA with the first resonant frequency at 2.5 GHz and a cylindrical acrylic liquid container were designed. The effects of high loss tangent of test liquids, such as ethanol, methanol, and water, on the input reflection coefficient and input impedance of the scaled SR-CSRR-BCS slot-loaded MPA were investigated. Finally, a quarter-wave transformer was appended between the 50 Ω feedline and the slot-loaded patch to enhance impedance mismatching due to the high loss tangent of the MUT.

The measured PRFS of the first resonant frequencies for ethanol, methanol, and water were 8.07%, 16.4%, and 34.53%, respectively, and measured PRFSE for ethanol, methanol, and water were 2.82, 1.88, and 1.4, respectively, compared to the RS-loaded MPA. The measured sensitivities (%) for ethanol, methanol, and water were 1.33%, 0.78%, and 0.45%, respectively, which was higher than other antenna-based microwave sensors. 

The proposed SR-CSRR-BCS slot-loaded MPA can be used to sense various types of micro-volume liquid chemicals or oils. It also might be used as a glucose concentration sensor in healthcare applications. An antenna is a one-port device using an input reflection coefficient characteristic that can take measurements with only a single port of the VNA. Therefore, it would be useful to make a simple, easy-to-operate, fast-to-prepare, and real-time measurement system compared to a conventional transmission line or filter- type system using two ports of the VNA.

## Figures and Tables

**Table 1:** Design parameters of the rectangular MPA loaded with four different types of slots.

Parameter	Value (mm)	Parameter	Value (mm)
*W*_g_	80	*w*_is_	2.8
*L*_g_	80	*l*_is1_	13
*W*	40	*g*_1_	1
*L*	31.9	*g*_2_	1
*w*_f_	1.66	*l*_is2_	9
*l*_f_	24.5	*l*_is3_	9
*l*_s1_	38	*l*_s2_	34
*w*_o_	1	*l*_is4_	7
*w*_s_	1	*h*	0.76

**Table 2:** Values of the first four resonant frequencies of the reference and four slot-loaded MPAs when varying the relative permittivity of the MUT superstrate from 1 to 10 (in gigahertz).

MPA Type		*ε*_r_ = 1	*ε*_r_ = 2	*ε*_r_ = 4	*ε*_r_ = 6	*ε*_r_ = 8	*ε*_r_ = 10
Reference	*f*_r1_	2.5	2.468	2.418	2.376	2.338	2.306
	*f*_r2_	4.046	3.994	3.93	3.874	3.824	3.778
	*f*_r3_	4.766	4.712	4.626	4.556	4.492	4.434
	*f*_r4_	5.148	5.048	4.902	4.778	4.664	4.574
RS-loaded	*f*_r1_	2.026	1.93	1.776	1.66	1.566	1.49
	*f*_r2_	2.752	2.69	2.6	2.532	2.476	2.43
	*f*_r3_	4.118	4.066	3.984	3.914	3.85	3.792
	*f*_r4_	5.136	5.078	4.968	4.872	4.774	4.674
SR-CSRR-BCS slot- loaded	*f*_r1_	1.8	1.678	1.494	1.362	1.26	1.18
	*f*_r2_	2.08	1.998	1.88	1.788	1.714	1.65
	*f*_r3_	3.088	3.016	2.898	2.806	2.728	2.662
	*f*_r4_	4.056	4.006	3.918	3.844	3.772	3.696
SR-CSRR-TCS slot- loaded	*f*_r1_	1.168	1.116	1.032	0.97	0.918	0.874
	*f*_r2_	2.744	2.642	2.45	2.286	2.15	2.036
	*f*_r3_	3.272	3.12	2.94	2.832	2.754	2.69
	*f*_r4_	4.08	4.026	3.938	3.868	3.804	3.746
DR-CSRR slot-loaded	*f*_r1_	0.896	0.858	0.798	0.75	0.712	0.678
	*f*_r2_	1.624	1.538	1.416	1.326	1.256	1.196
	*f*_r3_	2.344	2.182	1.946	1.78	1.656	1.56
	*f*_r4_	3.344	3.274	3.152	3.034	2.872	2.708

**Table 3:** Relative permittivity and loss tangent values of ethanol, methanol, and water at 2.5 GHz and 25 °C.

MUT	Relative Permittivity (*ε*_r_)	Loss Tangent (tan *δ*)
ethanol	7.08	0.965
methanol	22.15	0.596
water	77.7	0.12

**Table 4:** Sensitivity (%) comparison with other microwave sensors in the literature.

Resonator Type	Liquid Container Type	Liquid Volume (microliters)	Resonant Frequency (*f*_r_, GHz)	Sensitivity (%)	
MOCSRR in a CPW transmission line	Substrate integrated slot container	80	0.33	0.504	[13]
DR-CSRR slot-loaded MPA	Microfluidic channel	4	4.16	0.291	[22]
EMSIWA	Microfluidic channel	-	4.69	0.154	[28]
Cylindrical DRA	Two cylindrical microwells	150.8	5.432	0.172	[30]
SR-CSRR-BCS slot-loaded MPA	Cylindrical container	15	2.653	0.45	This Work

## Data Availability

Not applicable.
